# The compound LG283 inhibits bleomycin-induced skin fibrosis via antagonizing TGF-β signaling

**DOI:** 10.1186/s13075-022-02773-2

**Published:** 2022-04-29

**Authors:** Akira Utsunomiya, Takenao Chino, Hiroshi Kasamatsu, Takumi Hasegawa, Natsuko Utsunomiya, Vu Huy Luong, Takashi Matsushita, Yoko Sasaki, Dai Ogura, Shin-ichiro Niwa, Noritaka Oyama, Minoru Hasegawa

**Affiliations:** 1 Department of Dermatology, Division of Medicine, Faculty of Medical Sciences, University of Fukui, 23-3, Matsuokashimoaizuki, Eiheiji-cho, Yoshida-gun, Fukui, 910-1193 Japan; 2 Department of Dermatology, Hanoi Medical University, Hanoi, Vietnam; 3 Department of Dermatology, Faculty of Medicine, Institute of Medical, Pharmaceutical and Health Sciences, Kanazawa University, Kanazawa, Ishikawa, Japan; 4 Link Genomics, Inc., Chuo, Tokyo, Japan

**Keywords:** Systemic sclerosis, Fibrosis, Vascular injury, Therapy, Mouse model, TGF-β, EMT, Snail

## Abstract

**Background:**

Systemic sclerosis (SSc) is a collagen disease that exhibits intractable fibrosis and vascular injury of the skin and internal organs. Transforming growth factor-β (TGF-β)/Smad signaling plays a central role in extracellular matrix (ECM) production by α-SMA-positive myofibroblasts. Myofibroblasts may be partially derived from various precursor cells in addition to resident fibroblasts. Recently, our high-throughput in vitro screening discovered a small compound, LG283, that may disrupt the differentiation of epithelial cells into myofibroblasts. This compound was originally generated as a curcumin derivative.

**Methods:**

In this study, we investigated the effect of LG283 on inhibiting fibrosis and its mechanism. The action of LG283 on TGF-β-dependent fibrogenic activity and epithelial-mesenchymal transition (EMT) was analyzed in vitro. The effects of LG283 were also examined in a bleomycin-induced skin fibrosis mouse model.

**Results:**

LG283 suppressed TGF-β-induced expression of ECM, α-SMA, and transcription factors Snail 1 and 2, and Smad3 phosphorylation in cultured human dermal fibroblasts. LG283 was also found to block EMT induction in cultured human epithelial cells. During these processes, Smad3 phosphorylation and/or expression of Snail 1 and 2 were inhibited by LG283 treatment. In the bleomycin-induced skin fibrosis model, oral administration of LG283 efficiently protected against the development of fibrosis and decrease of capillary vessels without significantly affecting cell infiltration or cytokine concentrations in the skin. No apparent adverse effects of LG283 were found. LG283 treatment remarkably inhibited the enhanced expression of α-SMA and phosphorylated Smad3, as well as those of Snail 1 and 2, in the bleomycin-injected skin.

**Conclusions:**

The LG283 compound exhibits antagonistic activity on fibrosis and vascular injury through inhibition of TGF-β/Smad/Snail mesenchymal transition pathways and thus, may be a candidate therapeutic for the treatment of SSc. Although the involvement of EMT in the pathogenesis of SSc remains unclear, the screening of EMT regulatory compounds may be an attractive approach for SSc therapy.

**Supplementary Information:**

The online version contains supplementary material available at 10.1186/s13075-022-02773-2.

## Introduction

Systemic sclerosis (SSc) is an autoimmune disease characterized by tissue fibrosis caused by excessive deposition of collagen and other extracellular matrix (ECM) components in the skin and visceral organs [1–4]. Activated fibroblasts and α-smooth muscle actin (α-SMA)-positive myofibroblasts are mainly responsible for the excessive synthesis and tissue deposition of ECM in SSc. Fibroblast activation and the phenotypic transition towards myofibroblasts result from a complex series of events initiated by various pro-fibrotic molecules, including transforming growth factor-β (TGF-β), connective tissue growth factor (CTGF), platelet-derived growth factor (PDGF), and interleukin (IL)-4, IL-6, IL-13, IL-17A, and endothelin-1. Among them, TGF-β is likely the key molecule for functional activation of local fibroblasts and resultant tissue fibrosis in SSc [4, 5].

The binding of activated TGF-β to its cell surface receptor triggers intracellular signal transduction of canonical Smad-dependent and noncanonical Smad-independent pathways. In the Smad-dependent pathway, the activation of TGF-β receptor type I leads to phosphorylation of Smad2 and 3, allowing it to complex with Smad4 and translocate into the nucleus where it binds to Smad-binding element sequences of TGF-β responsive genes. Cofactors such as p300 are then recruited to the Smad-binding element-Smad complex, followed by transcriptional activation of the targeted genes. Excessive or dysregulated TGF-β/Smad signaling can result in ECM deposition and tissue fibrosis. Indeed, sustained signal activation has been detected in dermal fibroblasts of bleomycin-induced SSc mice [6] and inversely, blocking of the signaling cascade ameliorates skin fibrosis in experimental SSc models [7–11]. However, no established TGF-β-targeted therapy has successfully been translated to SSc patients.

TGF-β is well known to stimulate epithelial cells to undergo an epithelial-mesenchymal transition (EMT) in vitro [12, 13]. Snail family zinc finger proteins are direct targets of the TGF-β cascade in epithelial cells and play a critical role in EMT during development, carcinogenesis, and tissue repair [14]. Such an increase in gene expression caused by EMT-inducing transcription factors may thus promote some differentiation towards an EMT process in the SSc epithelium. However, a recent single-cell RNA-sequence investigation has demonstrated that dermal myofibroblasts are mainly derived from dermal fibroblasts in patients with SSc [15]. Nonetheless, the SSc epidermis shows active TGF-β signaling and increased SNAIL1 mRNA reflecting partially evoked EMT [16]. Therefore, epidermal cells acquire some mesenchymal phenotype and may contribute to skin fibrosis without fully transforming into myofibroblasts. Moreover, approaches to disrupt the process of EMT may be effective for the treatment of SSc via inhibiting the transition of fibroblasts and other precursor cells into myofibroblasts through a similar mechanism.

Our high-throughput screening system identified LG283 from more than 1200 compounds due to its ability to inhibit the development of mesenchymal features in human epithelial cell lines. This compound was originally generated as a curcumin derivative and has been reported as a tau aggregation inhibitor by the name of PE859 [17]. A previous paper demonstrated that this curcumin derivative is more effective than curcumin itself at inhibiting amyloidβ production [18]. Therefore, this compound may be potentially useful for the treatment of Alzheimer’s disease. There are also many reports that demonstrate the anti-fibrotic activity of curcumin via inhibition of TGF-β/Smad signaling in various organs [19]. Curcumin has been reported to inhibit TGF-β/Smad signaling via suppressing degradation of the TGF-induced factor, a negative regulator of TGF-β signaling, in SSc fibroblasts [20]. However, the in vivo effects of curcumin or its derivatives on SSc have not been demonstrated.

In this study, we show that LG283 exhibits suppressive effects on fibrosis in both cultured human dermal fibroblasts and in a mouse model. Our findings indicate that LG283 inhibits fibrosis mainly by antagonizing the TGF-β/Smad3/Snail pathway, which results in the augmentation of mesenchymal features of fibroblasts and epithelial cells.

## Materials and methods

### LG283

LG283 was designed and synthesized as one of a series of curcumin derivatives with the original name of PE859 (3-[(1E)-2-(1*H*-indol-6-yl)ethenyl]-5-[(1E)-2-[2-methoxy-4-(2-pyridylmethoxy) phenyl] ethenyl]-1*H*-pyrazole) by Okuda and Sugimoto et al. [18].

### Cell culture

Normal human dermal fibroblasts were purchased (Clontech) and were grown in Dulbecco’s modified Eagle’s medium (DMEM, Nacalai tesque) containing 10% fetal bovine serum (FBS), 100 U/ml penicillin, and 100 μg/ml streptomycin (Nacalai tesque) at 37 °C in a humidified 5% CO_2_ atmosphere. When cells reached ~70% confluency they were starved in DMEM containing 0.1% FBS for 24 h and then pretreated with dimethyl sulfoxide (DMSO) as a control or 4.5 μM of DMSO-diluted LG283. The LG283 doses were optimized on the basis of sequential pilot experiments (data not shown). One-hour after stimulation cells were stimulated with 10 ng/ml human recombinant (r) TGF-β1 (Peprotech) for use in experiments of immunofluorescence staining, real-time reverse transcription-polymerase chain reaction (RT-PCR), and western blot analysis. Fibroblasts between passages 3 and 5 were prepared for all experiments.

### EMT

The A549 human non-small cell lung carcinoma cell line (American Type Culture Collection) was maintained in DMEM supplemented with 10% heat-inactivated FBS. Cells were seeded in 96-well plates at a density of 10,000 cells/well, 384-well plates at a density of 3000 cells/well, and 3D-Nano-Culture Plates (SCIVAX Life Sciences). EMT was induced in DMEM containing 5% FBS with 5 ng/ml human rTGF-β2 (R&D Systems) for each indicated interval, with or without an incubation with LG283 (0.5μM) as described previously [21].

### Animal studies

Healthy female C57BL/6 mice aged 8–10 weeks (CLEA Japan), not just siblings, were used for a bleomycin-induced skin fibrosis model [22]. Bleomycin (1 mg/ml in saline) or 0.9% NaCl was injected subcutaneously into the shaved back of the mice (150 μl in each injection), concurrent with daily oral gavage of either LG283 (40 mg/kg or 80 mg/kg in sterilized olive oil) or olive oil alone for 4 weeks. The LG283 doses were optimized on the basis of sequential pilot experiments (data not shown). The drug was administered at the same time in all treatment groups for each independent experiment. All mice were housed in the same room of a specific pathogen-free barrier facility and screened regularly for pathogens.

### Immunofluorescence staining

After stimulating cells with 10 ng/ml rTGF-β1 for 2 h, they were washed twice in ice-cold PBS, fixed in 100% ethanol for 10 min at room temperature, and permeabilized with 0.1% Triton X-100 in PBS for 3 min. Cells were blocked with 2% FBS for 15 min, incubated with anti-phospho-Smad3 (p-Smad3) rabbit antibody (1:50 in 2% FBS; Cell Signaling Technology) for 60 min at room temperature and then with Alexa fluor 488-conjugated goat anti-rabbit antibody for 40 min. Coverslips were mounted using Vectashield with 4′,6-diamidino-2-phenylindole (DAPI, Vector Laboratories).

### RT-PCR

The total RNA was isolated from the skin or cultured fibroblasts using RNeasy spin columns (Qiagen) and was digested with DNase I (Qiagen) to remove chromosomal DNA. The total RNA was reverse-transcribed to a complementary DNA with random hexamers (Takara Bio). Real-time RT-PCR was performed using the StepOnePlus Real-Time PCR system (Applied Biosystems). All data were normalized against GAPDH mRNA and are expressed as relative expression.

### Western blot analysis

The total protein was extracted from human dermal fibroblasts or A549 human non-small cell lung carcinoma cell line using a 101 Bio Cytoplasmic & Nuclear Protein Extraction kit (Medibena). Samples of bleomycin-injected skin were homogenized in 600 μl of lysis buffer (10 mmoles/liter phosphate-buffered saline, 0.1% SDS, and 1% Nonidet P40, 5 mmoles/liter EDTA containing complete protease inhibitor mixture [Roche Diagnostics]) to extract proteins. Protein concentration was quantitated using a BCA protein assay kit (Takara Bio) on a spectrophotometer. An equal amount of protein was subjected to a standard SDS-PAGE and was transferred to a nitrocellulose membrane (Bio-Rad Laboratories Inc.). The blotted membrane was blocked for 30 min at room temperature with 5% skim milk/TBS, followed by incubation with anti-human antibodies to collagen type I alpha 2 chain (Col1A2, Abcam), fibronectin-1 (FN-1, LS bio), α-SMA (Abcam), E-cadherin (Abcam), Smad3 (Cell Signaling Technology), phospho-Smad3 (p-Smad3, Cell Signaling Technology), SNAIL1 (Gene Tex), SNAIL2 (Invitrogen), ZEB1 (Abcam), ZEB2 (Gene Tex), Twitst 1 (Santa Cruz biotechnology), GAPDH (Thermo Fisher Scientific), TUBULIN (Bio-Rad Laboratories Inc.) or anti-mouse antibodies to α-SMA (Abcam), Smad3 (Invitrogen), p-Smad3 (Invitrogen), and GAPDH (Sigma-Aldrich) overnight at 4 ^o^C. After washing with TBS-T three times, the membrane was incubated for 1 h at room temperature with an HRP-conjugated secondary antibody. The protein bands were visualized using Chemi-Lumi One Super solution (Nacalai tesque). All data were normalized against GAPDH or TUBULIN expression and are expressed as relative expression.

### Histologic analysis

Paraffin-embedded mouse skin sections (6 μm in thickness) were subjected to hematoxylin-eosin and Masson’s trichrome staining. For evaluation of skin fibrosis, the dermal thickness was defined computationally as the skin thickness from the epidermal-dermal junction to the junction between the dermis and subcutaneous fat [23]. Data were assessed in five distinct fields under an equal magnification (×40) using a light microscope and are expressed as mean **±** SEM. Each section was examined independently by two investigators (T.C. and N.O.) in a blinded manner. Collagen deposition was quantified on Masson’s trichrome-stained sections as the ratio of blue-stained area to total stained area using Adobe Photoshop Elements version 12.

### Immunohistochemical staining

Paraffin-embedded mouse skin sections (6 μm in thickness) were incubated for 120 min at room temperature with monoclonal antibodies (mAbs) to CD3 (1:200; Nichirei Bioscience), F4/80 (1:100; Abcam), α-SMA (1:200; Abcam), CD31 (1:200; Abcam), Snail1 (1:100, Gene Tex), Snail2 (1:100; Invitrogen), or cytokeratin 5 (1:500; BioLegend), then with HRP-labeled secondary antibody (Nichirei BioScience), followed by color development with the aminoethyl carbazole system (Nichirei BioScience). CD3^+^ cells and F4/80^+^ cells were counted under a high-power microscopic field (CD3^+^ cells and F4/80^+^ cells in three distinct fields). Each section was examined independently by two investigators (T.C. and N.O.) in a blinded manner. The area of the CD31-positive vessels, SNAIL1, or SNAIL2 was quantified as pixel counts in three distinct fields of each three skin sections using Adobe Photoshop Elements version 12.

### Immunofluorescence staining of mouse skin

Frozen skin sections (4 μm) were fixed for 10 min in ice-cold ethanol. After washing with PBS, sections were blocked by 4% Blocking ace (DS Pharma Biochemical) for 1 h at room temperature followed by incubating overnight at 4 ^o^C with primary antibodies to CD31 (1:200; Abcam), F4/80 (1:100; Abcam), or Snail1 (1:100, Gene Tex). Sections were then washed with PBS and incubated with species-specific secondary antibodies for 1 h at room temperature. Sections were mounted in VECTASHIELD mounting media containing 4′,6-diamino-2-phenylindole (Vector laboratories). Slides were visualized using laser scanning confocal microscopy (OLYMPUS FV1200).

### Cytometric bead array and ELISA

Concentrations of cytokines in the skin were determined by using the Cytometric Bead Array Mouse Inflammation Kit (BD Biosciences). The concentration of TGF-β1 in the skin was measured by enzyme-linked immunosorbent assay (ELISA; R&D Systems), according to the manufacturer’s instructions. Samples (40 mg) of bleomycin-injected skin were homogenized in 600 μl of lysis buffer (10 mmoles/liter phosphate-buffered saline, 0.1% SDS, 1% Nonidet P40, and 5 mmoles/liter EDTA containing complete protease inhibitor mixture [Roche Diagnostics]) to extract proteins. Homogenates were centrifuged at 15,000 revolutions per minute for 15 min at 4 °C to remove debris, and the supernatants were used for the measurement of cytokines.

### Preparation of skin cell suspensions

A 2×2.5-cm piece of depilated back skin was minced and digested in 7 ml of RPMI 1640 containing 10% FBS with 2 mg/ml crude collagenase (Sigma-Aldrich), 1.5 mg/ml hyaluronidase (Sigma-Aldrich), and 0.03 mg/ml DNase I (Roche Applied Science) at 37 ^o^C for 90 min. The samples were passed through a 70-μm Falcon cell strainer (BD Biosciences) to obtain single-cell suspensions. After centrifugation at 1500 rpm for 5 min, the cell pellet was resuspended in a 70% Percoll solution (GE Healthcare) and then overlaid with a 37% Percoll solution, followed by centrifugation at 1800 rpm for 20 min. The cells were aspirated from the Percoll interface and passed through a 70-μm Falcon cell strainer. The harvested cells were washed with ice-cold PBS and were used for flow cytometric analysis.

### Flow cytometry

mAbs used were Alexa fluor 488-conjugated anti-CD45, pacific blue-conjugated anti-CD11b, PerCP-conjugated anti-Ly6C, and APC-conjugated anti-CD204 (R&D Systems). To distinguish dead cells from live cells, the Live/Dead Fixable Aqua Dead Cell Stain kit (Invitrogen) was used. The single-cell suspensions obtained above were stained for 20 min at 4 ^o^C using indicated mAbs at predetermined optimal concentrations for 6-color immunofluorescence analysis. Stained samples were assessed using a FACSCanto II (BD Biosciences) followed by data analysis using FlowJo software version 7 (Ashland).

### Statistical analysis

All data were analyzed using Graphpad Prism software version 7 and are expressed as mean ± SEM. The significance of differences between samples was determined with Student’s 2-tailed *t*-test. *P*-values less than or equal to 0.05 were considered as statistically significant.

## Results

### LG283 inhibits TGF-β-induced expression of ECM in human dermal fibroblasts

**Fig. 1:**
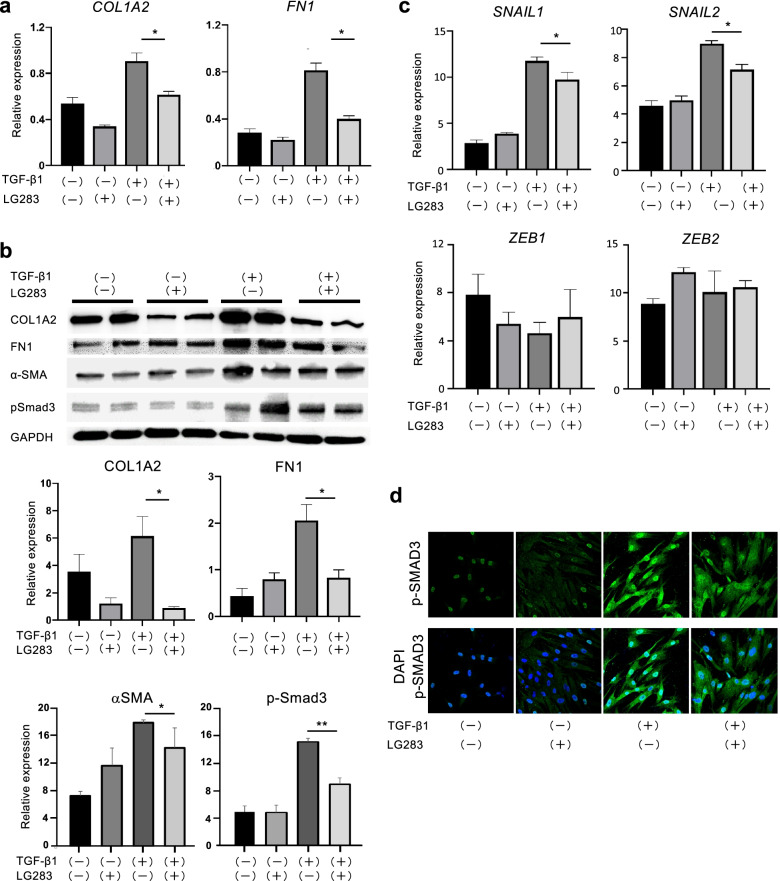
LG283 inhibits the fibrogenic activity of cultured human dermal fibroblasts stimulated with TGF-β1. Human fibroblasts were pretreated with DMSO or 4.5 μM of DMSO-diluted LG283 for 1 h, followed by stimulation with 10 ng/ml human rTGF-β1 for an additional 24 h. **a**, **b** After harvest, mRNA and protein expression of indicated molecules were evaluated by real-time RT-PCR and western blotting, respectively. Values were normalized to GAPDH levels and are shown as the mean of fold change compared to vehicle control ± SEM. Molecular weights of the paneled proteins; 130-kDa (COL1A2), 250-kDa (FN1), 50-kDa (α-SMA), 55-kDa (pSmad3), and 37-kDa (GAPDH). All values represent mean ± SEM; *n* = 5 each group; *p ≤ 0.05; **p ≤ 0.01. **c** Effects of LG283 on mRNA expression of transcription factors associated with the mesenchymal transition in human dermal fibroblasts. All values represent mean ± SEM; *n* = 5 each group; *p ≤ 0.05. **d** Human dermal fibroblasts were immunostained for phospho-Smad3 (p-Smad3, *green*). Nuclear counter-staining was performed with DAPI. Representative images are shown on the *left* (40-fold magnification)

Excessive production of ECM by skin fibroblasts or myofibroblasts contributes to skin fibrosis. Therefore, we examined the biological effects of LG283, a small compound that was detected as a candidate for the antifibrotic drug by our high-throughput in vitro screening, on ECM synthesis by cultured human normal skin fibroblasts. As assessed by real-time RT-PCR, baseline mRNA expression of COL1A2 and FN-1 was significantly increased by subsequent treatment with rTGF-β1 (Fig. 1a). In contrast, 1-h pretreatment with LG283 significantly suppressed the TGF-β1-dependent induction of both mRNAs to steady-state levels. A similar trend was observed for the protein expression of COL1A2 and FN-1, as assessed by western blotting (Fig. 1b). These findings suggest that pretreatment with LG283 efficiently inhibits TGF-β-induced fibrogenic activity of skin fibroblasts.

### LG283 inhibits the TGF-β-dependent increase of transcription factors responsible for the mesenchymal transition in human dermal fibroblasts

The differentiation of fibroblasts into myofibroblasts is critical for local ECM production and resultant fibrosis in the skin. This was supported by our finding that pretreatment with LG283 inhibited the TGF-β1-dependent expression of a mesenchymal marker α-SMA in human normal skin fibroblasts (Fig. 1b). Therefore, we investigated the expression of representative transcription factors responsible for the differentiation into myofibroblasts (Fig. 1c). The mRNA expression of zinc-finger transcriptional regulators SNAIL1 and SNAIL2 was found to increase following treatment with rTGF-β1. However, pretreatment with LG283 significantly inhibited the TGF-β-dependent induction of both mRNAs. On the other hand, rTGF-β1 and/or LG283 did not alter the expression levels of ZEB1 and ZEB2, other zinc-finger transcriptional regulators associated with the transition into myofibroblasts, suggesting LG283 specifically inhibits the Snail signaling in the TGF-β-dependent mesenchymal transition cascade.

### LG283 abrogates TGF-β-dependent phosphorylation of Smad3 in human dermal fibroblasts

The TGF-β binding to its receptor induces the phosphorylation of Smad2/3 transcription factors upon canonical signaling. Phosphorylated Smad2/3 and cytoplasmic Smad4 intercommunicate to transfer the signal to the nucleus and result in the transcriptional gene regulation responsible for tissue fibrosis. We investigated the effects of LG283 on phosphorylation of Smad3, the key transcription factor of TGF-β signaling, in cultured human normal skin fibroblasts. On immunoblotting, treatment with rTGF-β1 caused the over-phosphorylation of Smad3, but it was inhibited by pretreatment with LG283 (Fig. 1b). Also, the immunocytochemical analysis revealed that treatment with rTGF-β1 increased cytoplasmic and nuclear staining for p-Smad3 (Fig. 1d). However, Smad3 phosphorylation was inhibited by pretreatment with LG283.

### LG283 blocks TGF-β-induced EMT in cultured A549 lung epithelial cells

**Fig. 2:**
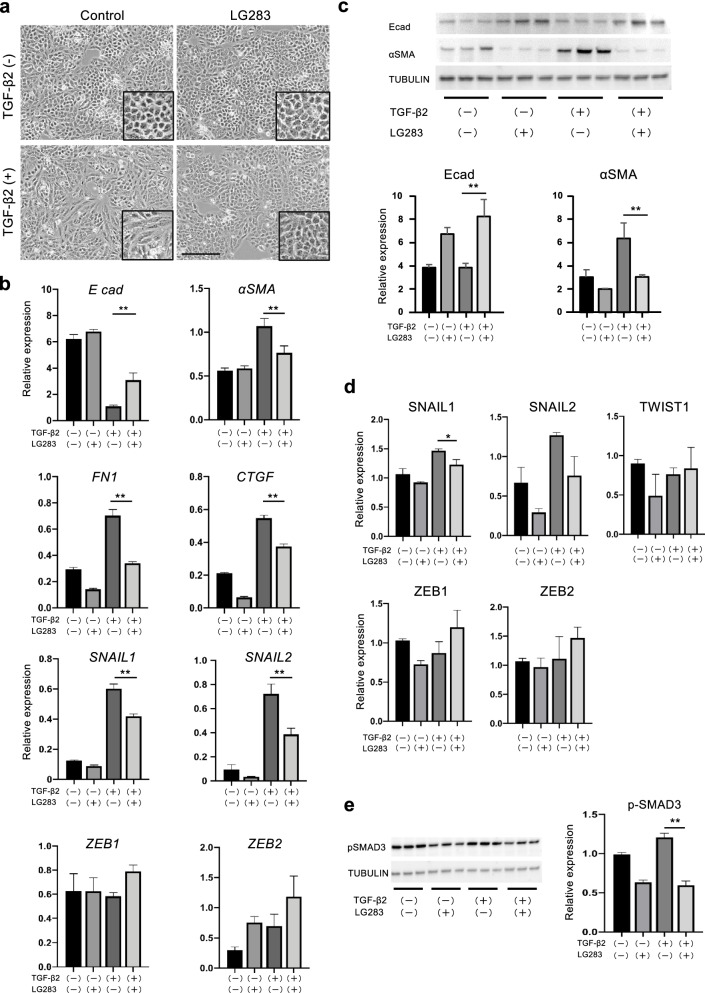
LG283 antagonizes EMT in vitro. The effect on EMT of human lung carcinoma epithelial cell line A549 cells in NanoCulture Plate (NCP). The cells were grown in either 2-D NCP conditions with or without 5 ng/ml rTGF-β2 only or TGF-β2-plus 0.5 μM of LG283. **a** Representative images of 2-D cultured A549 cells. The cells exhibited a round, confluent epithelioid appearance irrespective of the presence (*right upper*) or absence of LG283 (*left upper*). Treatment with rTGF-β2 for 72 h resulted in a morphological change to fibroblastic spindle shape (*left lower*), which was blocked by co-treatment with LG283 (*right lower*). *Inset* showed magnified images. Scale bars, 200 μm. **b** mRNA expression of epithelial (E-cadherin) and mesenchymal markers (FN-1, α-SMA, CTGF) and transcription factors associated with EMT (SNAIL1, 2 and ZEB1, 2) was quantified by real-time RT-PCR at 48h. Values were normalized to GAPDH levels and are shown as the mean of fold change compared to vehicle control ± SEM of three independent experiments; *n*=5 in each group; **p ≤ 0.01. **c**, **d** Protein expression of EMT-associated transcription factors was quantified by western blot analyses at 48–96h. Values were normalized to GAPDH or TUBULIN levels and are shown as the mean of fold change compared to vehicle control; *n*=3~4 in each group. Representative blot of α-SMA and E-cadherin is shown. All values represent mean ± SEM; *p ≤ 0.05; **p ≤ 0.01. Molecular weights of the paneled proteins; 98 kDa (Ecad), 48 kDa (α-SMA), and 50 kDa (TUBULIN). **e** After 48 h incubation of A549 cells with or without rTGF-β2 only or TGF-β2-plus LG283, protein expression of p-Smad3 was evaluated by western blotting. Values were normalized to TUBULIN levels and are shown as the mean of fold change compared to vehicle control ± SEM; *n*=4 in each group; **p ≤ 0.01. Molecular weights of the paneled proteins; 53 kDa (pSmad3) and 50 kDa (TUBULIN)

We performed an EMT assay using the A549 human lung carcinoma epithelial cell line. When cultured on 2-D plates, A549 cells rapidly grew to a confluent epithelioid sheet-like appearance, which was not affected by the presence of LG283 (Fig. 2a, *upper panels*). Morphologically, the cells appeared round with loose clusters and sparse intercellular adhesions. Upon treatment with rTGF-β2 for 72 h, the cells changed to a fibroblastic spindle shape (Fig. 2a, *left lower*). However, simultaneous treatment with rTGF-β2 and LG283 somewhat negated the morphological change of A549 cells (Fig. 2a, *right lower*).

The treatment with rTGF-β2 markedly reduced the expression of E-cadherin mRNA, a representative epithelial marker, but inversely increased the expression of mesenchymal markers such as FN-1, α-SMA, and CTGF at 48h (Fig. 2b). The altered expression pattern of epithelial and mesenchymal markers was significantly repressed by simultaneous treatment with LG283. Treatment with rTGF-β2 increased the mRNA expression of SNAIL1 and SNAIL2, but not that of ZEB1 and ZEB2 (Fig. 2b). The increased mRNA expression of SNAIL1 and 2 was suppressed by simultaneous treatment with LG283, although other transcription factors, ZEB1 and ZEB2, did not change in their mRNA expression (Fig. 2b). The protein expression of E-cadherin was increased, whereas protein levels of α-SMA were decreased in rTGF-β2-stimulated epithelial cells by simultaneous treatment with LG283 (Fig. 2c). Protein levels of SNAIL1 and 2 were increased by TGF-β2; however, the increase was reduced by simultaneous LG283 treatment (Fig. 2d). Protein levels of ZEB1 and 2 and TWIST1 were not significantly changed following treatment with TGF-β2 and/or LG283 at 96h (Fig. 2d). Furthermore, western blotting exhibited the antagonizing effect of LG283 on TGF-β1-dependent p-Smad3 expression (Fig. 2e). Thus, LG283 significantly blocks TGF-β-induced EMT via inhibition of Smad3 phosphorylation and subsequent Snail signaling in epithelial cells.

### LG283 inhibits the development of bleomycin-induced skin fibrosis in mice

**Fig. 3:**
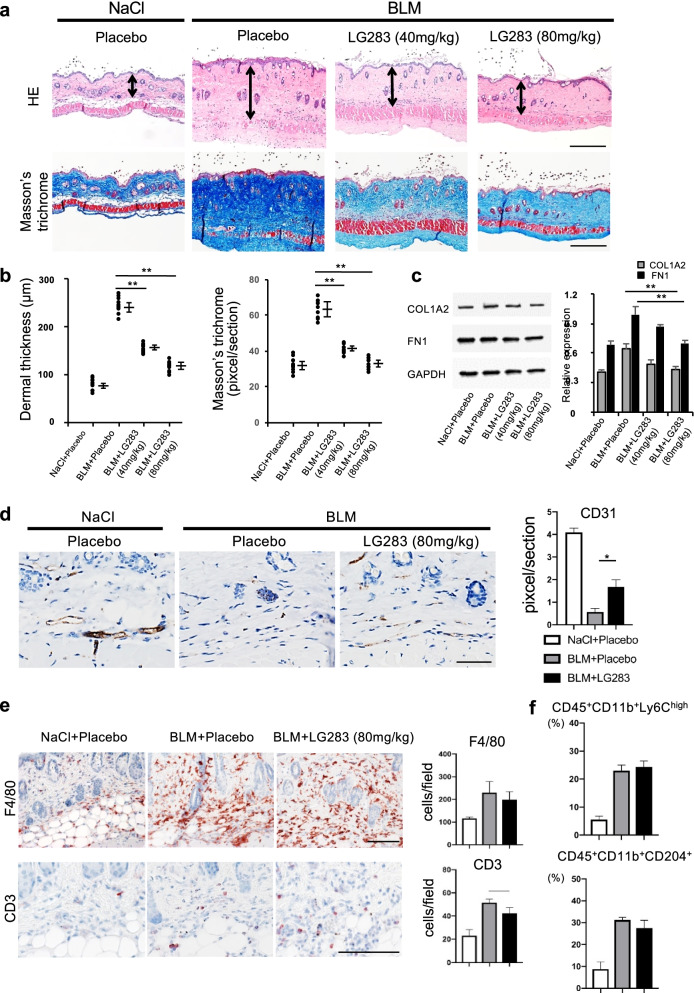
Oral LG283 administration ameliorates bleomycin-induced skin fibrosis regardless of inflammation in mice. a The antifibrotic effects of LG283 were analyzed in the back skin of mice receiving daily subcutaneous injections of bleomycin concurrent with daily oral gavage of LG283 or placebo (sterilized olive oil) for 4 weeks. Representative images of H&E stained (*upper columns*) and Masson’s trichrome stained tissue (*lower columns*). *Double-headed allows* indicate the measured length of dermal thickness. Scale bar, 200 μm. b Skin fibrosis in bleomycin-injected mice with or without oral LG283 was compared by determining dermal thickness and ratio of trichrome-stained area/total area. Values represent mean ± SEM; *n* =10 each group; **p ≤ 0.01. c Skin fibrosis in bleomycin-injected mice with or without oral LG283 was evaluated by expression of COL1A2 and FN1. Values were normalized to GAPDH levels and are shown as the mean of fold change compared to vehicle control; *n*=3 each group. All values represent mean ± SEM; **p ≤ 0.01. Molecular weights of the paneled proteins; 130 kDa (COL1A2), 250 kDa (FN1), and 37 kDa (GAPDH). d Oral LG283 suppressed the bleomycin-induced decrease of CD31-positive dermal capillary vessels in mice. Representative immunohistochemistry images of day 28 are shown (*left*). Scale bar, 50 μm. Quantitative analysis is shown in the bar graph (*right*). Values represent mean ± SEM; *n* =3 each group; *p ≤ 0.05. e Oral LG283 did not affect the infiltration of F4/80-positive or CD3-positive leukocytes in the skin of mice after 7 days of daily bleomycin injections. Representative images are shown (*left*). Scale bar, 50 μm. Quantitative analysis is shown in the bar graph (*right*). Values represent mean ± SEM; *n* =5 in each group. f Oral LG283 did not change the infiltration of inflammatory CD11b^+^Ly6C^hi^ monocytes and profibrotic CD11b^+^CD204^+^ M2 macrophages analyzed by flow cytometry after 21 days of bleomycin injection. Values represent mean ± SEM; *n* = 5 each group

Using a bleomycin-induced skin fibrosis mouse model, we examined the in vivo antifibrotic effects of LG283. Subcutaneous bleomycin injection and oral LG283 were co-administrated daily for 4 weeks. No apparent side effects including the change of body weight and activity were observed in any mice (data not shown). Histologically, skin thickness was increased more than two-fold following bleomycin injection, which was significantly reduced by both doses (40 mg/kg and 80 mg/kg) of oral LG283 (Fig. 3a, *upper columns* and b, *left*). Similarly, the Masson’s trichrome-stained area was significantly reduced in bleomycin-injected skin sections from LG283-treated mice, compared to those from mice treated with placebo (Fig. 3a, *lower columns* and b, *right*). These histological changes were consistent with the expression levels of COL1A2 and FN1 protein; that is, their protein levels were reduced with LG283 treatment (Fig. 3c).

### LG283 suppresses the reduction of the capillary vessels in the skin of bleomycin-treated mice

To investigate the effect of LG283 on vascular injury, the capillary vessels were stained with anti-CD31 antibody in bleomycin-injected skin on day 28. Subcutaneous bleomycin injection reduced the capillary vessels (Fig. 3d), similar to what is seen in the skin of SSc patients. However, simultaneous administration of oral LG283 significantly suppressed this decrease in capillary vessels in the skin (Fig. 3d). Thus, this suggests LG283 treatment is protective against destructive vascular injury during the process of skin fibrosis.

### LG283 does not affect inflammatory cell infiltration during the early-stage of bleomycin-induced skin fibrosis

Subcutaneous injection of bleomycin induces an early and transient inflammation mediated by locally infiltrating macrophages and other inflammatory cells [6]. Local injection of bleomycin, but not control saline, induces increased infiltration of F4/80-positive macrophages into the dermal and subcutaneous tissues at day 7 (Fig. 3e). In addition, there was evident local infiltration of CD3-positive T cells in bleomycin-injected skin, but not in control skin (*p*<0.05; Fig. 3e). However, oral LG283 administration did not affect the infiltration of these cell subsets.

To further characterize the macrophage subset present in the bleomycin-treated skin, we isolated CD11b-positve leukocytes from the lesional skin on day 21 and stained for monocyte/macrophage surface markers. As reported previously [24], proinflammatory macrophages (CD11b^+^Ly6C^hi^) and profibrotic M2 macrophages (CD11b^+^CD204^+^) were both increased in bleomycin-injected skin. Oral LG283 did not significantly reduce the infiltration of macrophage subsets (Fig. 3f). Thus, LG283 does not appear to significantly affect the skin inflammation induced by bleomycin injection.

### LG283 does not affect proinflammatory or profibrotic cytokine production, but remains alter the infiltrating α-SMA-positive cells in the bleomycin-injected skin

**Fig. 4:**
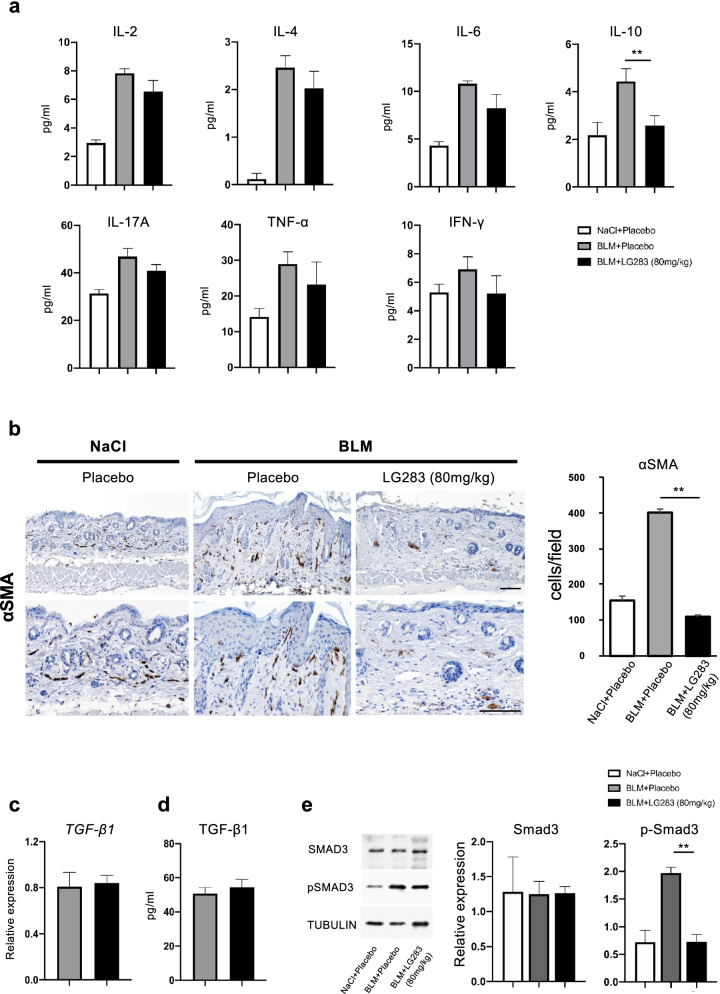
The effect of oral LG283 on the expression of cytokines and Smad3. Crude lysate and mRNA were extracted from skin samples of placebo- and LG283-treated mice on day 7 after bleomycin injection. **a** Concentration of the indicated cytokines was examined by cytometric bead array. Values represent mean ± SEM; *n*=5 each group; **p< 0.01. **b** αSMA expressing cells were quantified by immunohistochemistry. Representative images are shown (*left*). Scale bar, 50 μm. Quantitative analysis is shown in the bar graph (*right*). Values represent mean ± SEM; *n* =3 each group. **p< 0.01. **c**, **d** mRNA and protein concentration of TGF-β1 were evaluated with real-time RT-PCR of the skin and enzyme-linked immunosorbent assay of the skin extracts, respectively. Values represent mean ± SEM; *n* = 5 each group. **e** Protein expression levels of Smad3 and p-Smad3 were quantified using western blotting. Values were normalized to tubulin levels and were shown as the mean of fold change compared to vehicle control; *n*=3 each group. Values represent mean ± SEM; *n* = 5 each group; **p< 0.01. Molecular weights of the paneled proteins; 53 kDa (Smad3 and pSmad3) and 50 kDa (TUBULIN)

The process of early inflammation and subsequent fibrosis following subcutaneous bleomycin injection can be associated with the increased production of various proinflammatory and profibrotic cytokines. In general, the concentrations of investigated cytokines, IL-2, IL-4, IL-6, IL-10, IL-17A, TNF-α, and interferon (IFN) -γ, were increased in fluid from the bleomycin-injected skin on day 7 (Fig. 4a). Among these cytokines, the concentration of IL-10, a representative regulatory cytokine, in whole extracts from the bleomycin-injected skin was significantly reduced by co-administration of oral LG283. However, oral LG283 treatment did not significantly change the concentration of proinflammatory cytokines, such as IL-2, IL-6, IL-17A, TNF-α, and IFN-γ, or of a profibrotic cytokine, IL-4. Immunohistologically, the number of dermal α-SMA-positive-cells suggestive myofibroblasts was increased by subcutaneous bleomycin injection but turned to decrease with oral LG283 (Fig. 4b). Thus, LG283 inhibited mesenchymal differentiation without affecting the expression of key cytokines in the bleomycin-injected skin.

### LG283 antagonizes the expression of phosphorylated Smad3 in the bleomycin-injected skin

TGF-β/Smad signaling has been considered to be essential for tissue fibrosis. Therefore, the effect of LG283 treatment on TGF-β/Smad signaling was evaluated in the bleomycin-injected skin on day 7. The expression of TGF-β1 mRNA in the bleomycin-injected skin was not significantly affected by oral LG283 (Fig. 4c). Similarly, oral LG283 administration did not change the concentration of TGF-β1 protein in the bleomycin-injected skin extraction fluid (Fig. 4d). Expression levels of Smad3 protein were not affected by bleomycin and/or LG283 treatment (Fig. 4e). In contrast, expression of the p-Smad3 protein was markedly increased in bleomycin-injected skin compared to controls, an effect that was significantly inhibited by LG283 treatment of mice (Fig. 4e). Thus, LG283 treatment specifically inhibits the expression of the p-Smad3 in the skin of the bleomycin-induced skin fibrosis model.

### LG283 suppresses snail expression in bleomycin-injected skin

**Fig. 5:**
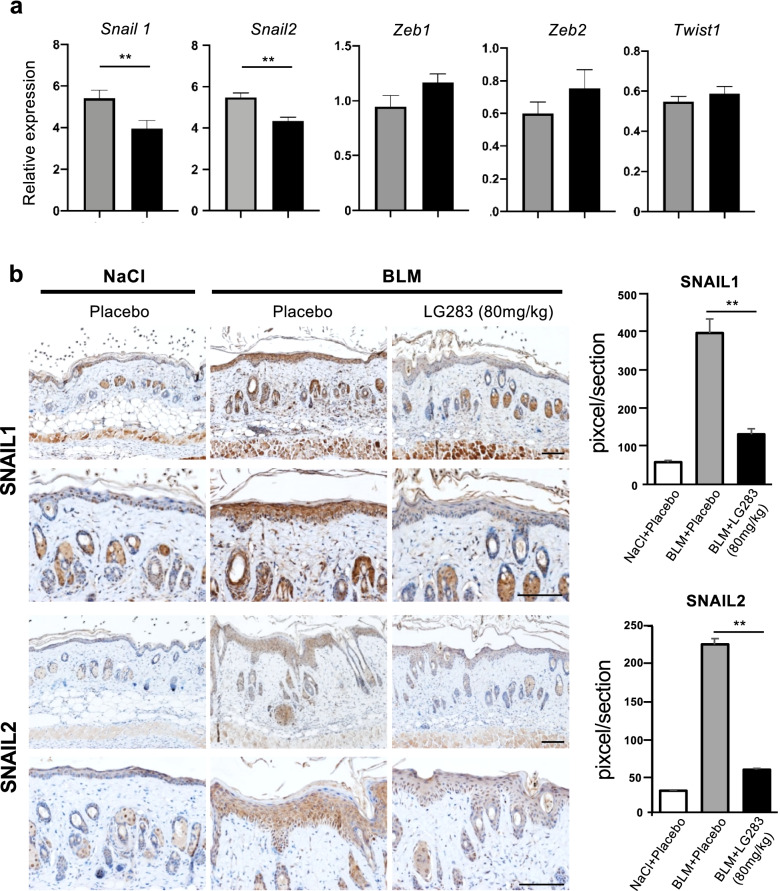
The effect of oral LG283 on the expression of transcription factors associated with EMT in bleomycin-injected mouse skin. **a** mRNA expression of indicated transcription factors in skin samples of placebo- and LG283-treated mice on day 7 after bleomycin injection were quantitatively analyzed by real-time RT-PCR. Values represent mean ± SEM; *n* = 5 each group; **p< 0.01. **b** Skin sections of placebo-and LG-283-treated mice on day 7 after bleomycin injection were immunostained for SNAIL1 and 2. Scale bar, 50 μm. Representative immunohistochemistry images are shown and staining-positive areas were compared among each group. Values represent mean ± SEM; *n* = 3 each group; **p< 0.01

Since our in vitro findings indicate that LG283 inhibits TGF-β-induced EMT, we examined the in vivo expression of transcription factors associated with EMT. Similar to what was seen in vitro, the expression of Snail1 and Snail2 mRNAs were significantly reduced in bleomycin-injected skin following administration of oral LG283 on day 7 (Fig. 5a). However, the expression of Zeb1, Zeb2, and Twist1 mRNAs were not significantly changed by oral LG283.

Consistent with these findings, immunohistopathology showed that the expression of Snail1 and 2 were augmented in bleomycin-injected skin on day 7 (Fig. 5b). However, oral LG283 inhibited the expression of both transcription factors in epidermal keratinocytes, follicular epithelial cells, dermal fibroblasts, and infiltrating cells (Fig. 5b). On the other hand, the expression of cytokeratin 5, a representative keratinocyte marker, in the epidermis was apparently unaffected by bleomycin injection and/or LG283 treatment (Supplemental Fig. 1). Thus, LG283 treatment specifically inhibits the expression of the Snail transcription factor in the skin following bleomycin treatment.

## Discussion

In this study, we investigated the inhibitory effects of the tau aggregation inhibitor, LG283, on skin fibrosis and vascular injury both in vitro and in vivo. LG283 disrupted the TGF-β-dependent increase of α-SMA, Smad3 phosphorylation, Snail1 and 2 expression, and major ECMs in cultured human skin fibroblasts and lung epithelial cells. Moreover, LG283 ameliorated skin fibrosis and vascular injury in a mouse model induced by subcutaneous injection of bleomycin. The in vivo effects of LG283 were largely attributable to the suppression of α-SMA, p-Smad3, and overexpression of Snail1 and 2. However, this compound did not affect inflammatory cell infiltration or concentration of major proinflammatory and/or profibrogenic cytokines in the skin during the fibrogenic process. Our results illustrate the antagonistic effects of LG283 and its potential for therapeutic application for inhibition of mesenchymal differentiation and the fibrogenic response.

Dermal fibroblasts from SSc skin show constitutive phosphorylation and nuclear translocation of Smad2/3 with various levels of activated Smad signaling. Therefore, targeting the TGF-β/Smad signaling is an attractive strategy for the treatment of SSc. In the current study, we confirmed that LG283 inhibits the expression of COL1α and FN-1 in human dermal fibroblasts stimulated with TGF-β1. During the process, LG283 blocks phosphorylation of Smad3, a representative receptor-regulated Smad, and expression of Snail 1 and 2, major downstream transcription factors specific for the mesenchymal phenotype. Thus, LG283 shows anti-fibrotic activity via suppression of TGF-β/Smad/Snail signaling in dermal fibroblasts. Nonetheless, the effect of LG283 for other Smads and Smad-independent non-canonical pathway has not been denied.

Profibrotic myofibroblasts may be partially derived from various precursors including pericytes, bone marrow-derived circulating cells, epithelial cells, endothelial cells, and adipocytes [25–27], although a recent study demonstrated that most dermal myofibroblasts are derived from dermal fibroblasts in SSc [15]. Among them, the transition from epithelial cells into myofibroblasts has not been clearly demonstrated in the skin of SSc or its animal model. However, we focused on EMT, which is relatively easy to induce in vitro in the current study. LG283 showed in vitro suppressive effects on EMT of human lung epithelial cells induced by TGF-β2. Additionally, LG283 inhibited the expression of p-Smad3 in lung epithelial cells stimulated with TGF-β2. Among various cytokine-transcriptional cascades involved in the process of EMT, Snail1 and 2 are dominant downstream transcription factors of TGF-β/Smad signaling and are likely to play central roles for EMT. LG283 significantly inhibited the expression of both Snail 1 and Snail 2. Moreover, TGF-β1 stimulation did not increase the expression of Zeb 1 or 2, and the expression levels of these genes were not affected by LG283 treatment suggesting the mechanism is specific. These findings indicate that LG283 may antagonize Smad3 phosphorylation downstream of TGF-β as well as the subsequent upregulation of Snail1 and 2 in fibroblasts and epithelial cells during the fibrotic process.

Consistent with these in vitro findings, oral LG283 administration was also effective against skin fibrosis and destructive vascular injury in a bleomycin-induced skin fibrosis mouse model. In this model, skin fibrosis is preceded by an increase of inflammatory cell infiltration and inflammatory cytokine expression [28]. However, LG283 did not affect the infiltration of CD3-positive T cells and macrophage subsets, including Ly6C^hi^ proinflammatory and CD204^+^ profibrotic macrophages, or the concentration of proinflammatory cytokines, including IL-2, IL-6, IL-17A, IFN-γ, tumor necrosis factor (TNF)-α, and profibrotic cytokine IL-4, in the inflammatory stage of bleomycin-injected skin. Therefore, the major anti-fibrotic effects of LG283 in bleomycin-injected skin do not appear to be mediated via suppression of inflammatory cell infiltration or subsequent cytokine production.

Since TGF-β/Smad3 signaling has been considered to contribute to the development of bleomycin-induced skin fibrosis [8–11, 29, 30], we evaluated the effect of LG283 on this signaling cascade. Although LG283 did not affect the expression of TGF-β1, increased expression of p-Smad3 was significantly inhibited by LG283 treatment in the bleomycin-injected skin. Interestingly, the expression of Snail1 and/or 2 was increased in skin cells including epidermal keratinocytes, fibroblasts, and infiltrating cells following bleomycin injection, an effect inhibited by LG283 treatment. These findings were similar to that of in vitro investigation of TGF-β-stimulated fibroblasts and epithelial cells. Therefore, our findings indicate that disrupted TGF-β/Smad/Snail signaling by LG283 may ameliorate skin fibrosis via antagonizing the differentiation of resident fibroblasts. Epithelial cells do not likely fully transform into myofibroblasts, but they may be involved in the development of fibrosis by incompletely differentiating into mesenchymal phenotype. The possibility that LG283 may have an inhibitory effect on the mesenchymal transition of other precursor cells needs further investigation. Especially, the action for endothelial cell mesenchymal transition (EndoMT) should be evaluated since LG283 prevented some degree of capillary loss in bleomycin-induced skin. EndoMT has been considered to be possibly important for the development of fibroproliferative and/or destructive vasculopathy and thereby may link the development of endothelial dysfunction/loss and skin fibrosis in SSc [27, 31].

A few other points remain unclear and future studies will be required to address these issues. The effects of LG283 on skin fibrosis have only been studied in one animal model. Additionally, the anti-fibrotic and anti-vasculopathic effects of LG283 in other organs will need to be investigated in animal models. Thus, additional preclinical investigations will be required, and the more precise safety profile of this compound will be needed to determine prior to the use of LG283 in SSc clinical trials.

## Conclusions

**Fig. 6:**
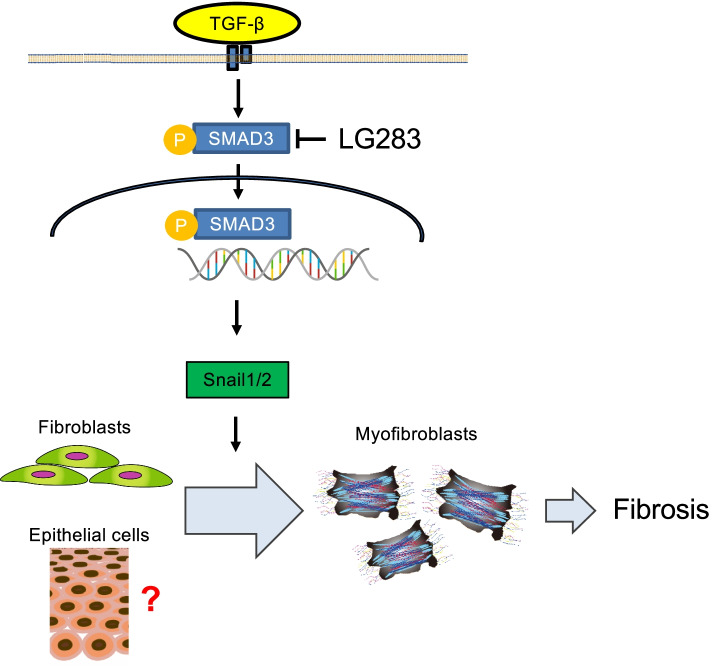
Putative mechanism of LG283-mediated inhibition of skin fibrosis

The small compound LG283 that has an inhibitory function for EMT in vitro may be effective for the treatment of skin fibrotic disorders mainly via inhibition of Smad3 phosphorylation and consequent Snail1 and 2 expression downstream of the TGF-β receptor in dermal fibroblasts. As a result, the differentiation of resident fibroblasts into myofibroblasts and subsequent tissue fibrosis are inhibited (Fig. 6). On the other hand, the transition of epithelial cells into mesenchymal cells and its blockade by LG283 remains unclear in vivo. Nevertheless, therapies that disrupt the process of EMT may be effective in fibrosis by blocking the transition of fibroblasts and other precursor cells into myofibroblasts in a similar mechanism. We propose that the screening of EMT regulatory compounds may result in additional therapeutic approaches for SSc treatment.

## Supplementary Information

**Additional file 1: Supplementary Figure 1.** Immunostaining for cytokeratin 5. Skin sections of placebo-and LG-283-treated mice on day 7 after bleomycin injection were immunostained for cytokeratin 5. Scale bar, 50 μm. Representative immunohistochemistry images are shown. n = 3 each group.

## Data Availability

The datasets used and/or examined for the current study will be available from the corresponding author on reasonable request.

## Abbreviations

SSc: Systemic sclerosis
TGF: Transforming growth factor
ECM: Extracellular matrix
α-SMA: α-Smooth muscle actin
EMT: Epithelial-mesenchymal transition
EndoMT: Endothelial cell mesenchymal transition
CTGF: Connective tissue growth factor
PDGF: Platelet-derived growth factor
IL: Interleukin
DMSO: Dimethyl sulfoxide
RT-PCR: Real-time reverse transcription-polymerase chain reaction
GAPDH: Glyceraldehyde-3-phosphate dehydrogenase
DMEM: Dulbecco’s modified Eagle’s medium
p-Smad3: Phospho-Smad3
Col1A2: Collagen type I alpha 2 chain
FN-1: Fibronectin-1
DAPI: 4′,6-Diamidino-2-phenylindole
TNF: Tumor necrosis factor
IFN: Interferon
