# Supplementary figures and images for: The compound LG283 inhibits bleomycin-induced skin fibrosis via antagonizing TGF-β signaling
Source: Arthritis Res Ther. 2022 Apr 29;24:94. doi: 10.1186/s13075-022-02773-2 (PMC9052694; doi:10.1186/s13075-022-02773-2)

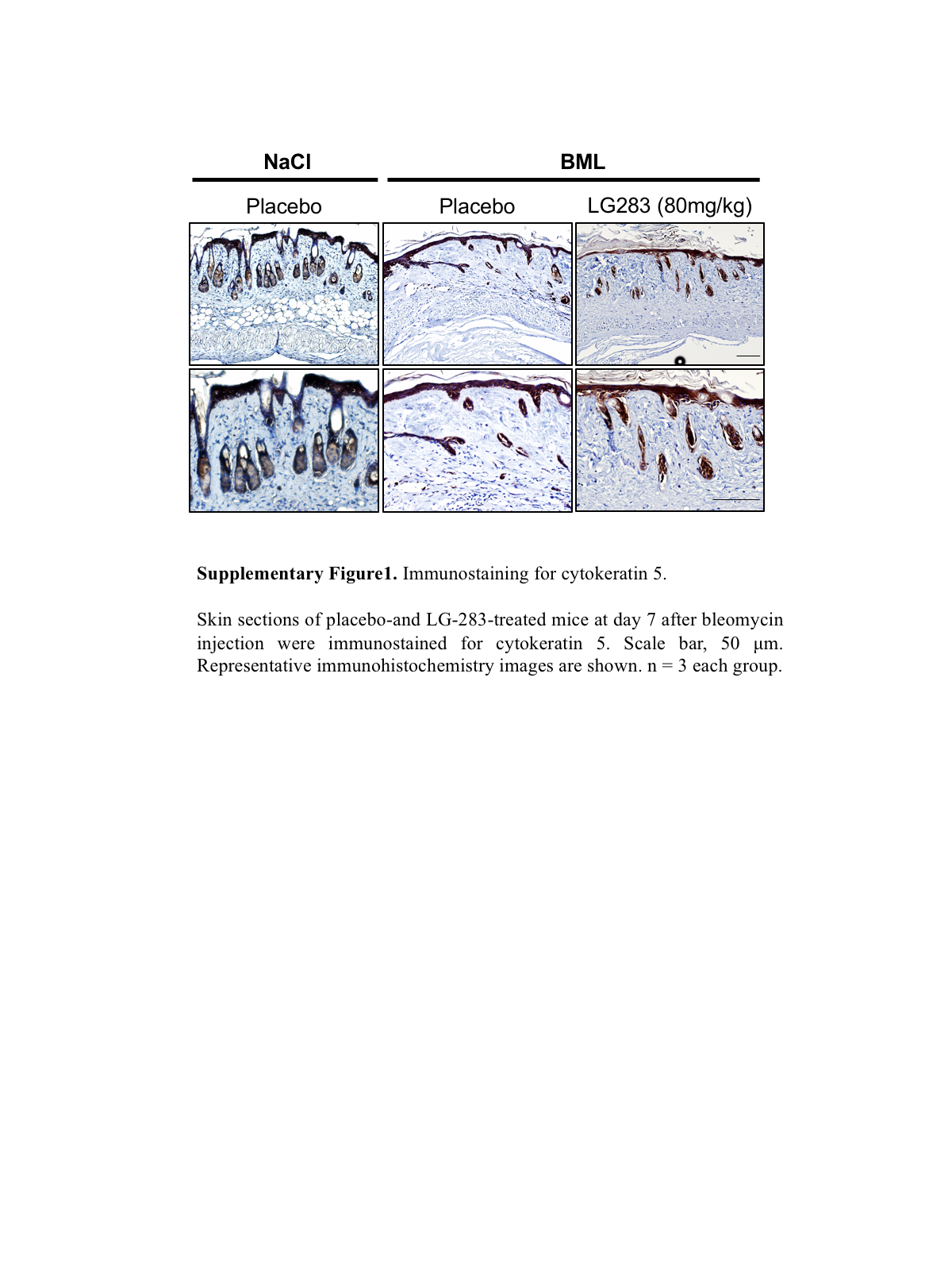

Supplement: Supplementary file 1 — Additional file 1: Supplementary Figure 1. Immunostaining for cytokeratin 5. Skin sections of placebo-and LG-283-treated mice on day 7 after bleomycin injection were immunostained for cytokeratin 5. Scale bar, 50 μm. Representative immunohistochemistry images are shown. n = 3 each group. [file 13075_2022_2773_MOESM1_ESM.tiff]
